# Regional Anesthesia in Cardiac Surgery: A Review of the Literature

**DOI:** 10.7759/cureus.18808

**Published:** 2021-10-15

**Authors:** Tianyu Jiang, Andrewston Ting, Michael Leclerc, Kerry Calkins, Jeffrey Huang

**Affiliations:** 1 Anesthesiology, Oak Hill Hospital, Brooksville, USA; 2 College of Medicine, University of South Florida, Tampa, USA; 3 Anesthesiology, HCA Healthcare, Orlando, USA

**Keywords:** review article, adult cardiac surgery, cardiac anaesthesia, thoracic anesthesiology, regional anesthesiology

## Abstract

With our population getting older and sicker, we are witnessing a steady increase in the volume of cardiothoracic procedures performed. As the role of anesthesiologists continues to shift towards being perioperative physicians, it is crucial to tailor the anesthetic to manage the surgical pain in both intraoperative and postoperative periods. In cardiac surgery, poorly controlled surgical pain can lead to opioid-induced hyperalgesia as well as chronic pain syndrome. As current practice encourages early extubation and decreased length of stay, clinicians have increasingly steered away from heavy intraop narcotic therapy over the past two decades. To blunt the sympathetic response and postoperative pain control, some have been using various fascial plane nerve blocks to reduce opioid use during surgery. These blocks are considered very safe to perform and do not lead to hemodynamic changes seen in neuraxial blockades. In this review article, we provide a brief overview of each of the commonly used blocks and summarize and discuss the latest clinical data for each of the common blocks and their efficacy in the setting of cardiothoracic surgery.

## Introduction and background

Cardiovascular disease is the leading cause of mortality worldwide, accounting for approximately one-third of all deaths [1]. The aging population in the United States will undoubtedly lead to an increase in the volume of cardiothoracic procedures [2]. Poorly controlled surgical pain is associated with the development of chronic pain in 20-50% of the patients following sternotomy and thoracotomy [3-5]. In the early 1990s, high-dose long-acting opiate anesthetic strategies were regularly utilized to blunt the sympathetic response from surgical pain and promote hemodynamic stability [6]. This resulted in patients requiring prolonged mechanical ventilation and thereby extending the time spent in the ICU. While opioid therapy is still the principal means of controlling pain following cardiac surgery, this practice has changed over the past two decades with the growing expectation towards “fast-tracking” with earlier extubation, reduced lengths of stay, and earlier discharges. Likewise, the current sociopolitical efforts in battling the opioid epidemic have made multimodal analgesia in cardiothoracic surgery even more appealing. With the increasing incorporation of ultrasound-guided regional anesthesia into the multimodal analgesia regimen and enhanced recovery after surgery (ERAS) protocols, it is no longer unusual to extubate immediately after surgery in the operating room [7].

While it is possible to use neuraxial techniques in cardiac surgery, the hemodynamic instability and potential for spinal hematoma make it controversial. Of note, the use of total spinal plus general anesthesia (GA) for cardiac surgery has been described in the literature without a single incidence of spinal hematoma [8]. The chest wall blocks such as pectoralis fascial (PECS), serratus anterior plane (SAP), erector spinae plane (ESP), and paravertebral (PVB) blocks are becoming attractive options since they do not result in hemodynamic changes as seen with neuraxial blockades. One of the major disadvantages of the chest wall blocks is the lack of effect on the internal mammary region, resulting in residual pain. The sternal blocks such as parasternal intercostal nerve blocks (PSINB) and thoracic transversus muscle plane block (TTMPB) have been devised to help with such shortcomings since they can reliably anesthetize the anterior branches of T2-T7 intercostal nerves [9].

The purpose of this review article is to provide a general overview of the chest wall and sternal blocks, as well as summarize and discuss the latest clinical data regarding each block and its clinical outcomes.

## Review

PECS I and II blocks

First described in 2011 and 2012, pectoralis I (PECS I) and pectoralis II (PECS II) blocks were used in breast surgeries to provide analgesia to the upper anterolateral chest wall [10,11]. Kumar et al. [12] randomized 40 patients undergoing coronary artery bypass grafting (CABG) or valve surgeries via midline sternotomy to postop PECS block or no block. The PECS group was extubated significantly earlier (p<0.0001). Pain scores at rest and with cough were also lower in the PECS group at zero, three, six, 12, and 18 hours after extubation (p<0.05). Additionally, peak inspiratory flow rates accessed by incentive spirometry were higher in the PECS group. Yalamuri et al. [13] have also described a case using PECS block as rescue analgesia in a patient undergoing mitral valve repair via right anterior thoracotomy approach. The block provided near-complete chest wall analgesia using 30 ml of 0.20% ropivacaine with 1:400,000 epinephrine.

Furthermore, in a study by Marcoe et al. [14], 112 patients receiving multimodal analgesia with either PECS 1 block, subcostal TAP (TAPPEC), or multimodal analgesia without any regional block were compared. The groups receiving the regional blocks required 51.1% less opioids intraoperatively (p<0.001) and 46.9% less overall (p<0.001). However, the differences in terms of postoperative opioid consumption and length of stay were not found to be statistically significant.

PECS blocks are considered very safe due to the lack of major neurovascular bundles surrounding the area of interest [15]. With ultrasound guidance, this block has a short learning curve. The current data clearly demonstrates earlier extubation and reduced intraoperative narcotic consumption [12,14], which are inherently beneficial for the patient. Adequate pain control is critical for respiratory mechanics and metabolic activity, especially for cardiac patients. Since this block is performed in the supine position, it is convenient to place and should not impact the logistics of the operating room workflow. In addition, it has great potential as a postop analgesic option for cardiac surgeries, possibly as a rescue block. The small sample size was a major limitation of the studies summarized.

Serratus anterior plane block

Serratus anterior plane (SAP) block was first described in 2013 to block thoracic intercostal nerves to provide analgesia to the lateral cutaneous branches of the intercostal nerves from T3 to T9 [16]. SAP is an extension of the PECS II block with an injection that is more inferolateral and has a wider spread. However, this block spares the mid-chest [16]. Some studies suggest that volumes greater than 40 ml are needed to achieve a spread that covers T1-T8 [17].

While the SAP block has been extensively described for thoracotomies, currently there are no studies on the use of SAP block for sternotomy. However, a few studies have indeed demonstrated its utility in cardiac device implantation procedures. De Waroux et al. [18] found that a single-shot SAP block allowed anesthesiologists to avoid GA and perioperative opioid use for cardiac defibrillator implantation. Droghetti et al. [19] replicated the same result in their study, except for one patient who required conversion to GA due to anxiety. Magoon et al. [20] randomized 100 adults undergoing cardiac surgery via thoracotomy approach to SAP, PECS II, or intercostal nerve block groups. They found that early pain scores were similar among all three groups, but late mean pain scores were significantly lower in SAP and PECS II groups (p<0.05). The amount of rescue fentanyl required was significantly higher in the intercostal group compared to SAP and PECS II groups (p<0.001).

Possible complications with SAP block include infection, pneumothorax, and local anesthetic toxicity due to the higher required volume of injection. However, SAP block is considered very safe to use in cardiothoracic procedures because the complications are exceedingly rare with the use of ultrasound guidance. Similar to the PECS block, SAP is usually performed in the supine position, which eliminates the additional need for patient repositioning. This block can be performed after the induction of GA, which can eliminate patient discomfort and anxiety. In addition, this block has almost no risk for pleural injury or spinal cord injury compared to the paravertebral block. The advantage over neuraxial techniques such as high spinal or thoracic epidural is that SAP should have very little to no hemodynamic effects due to the lack of sympathectomy. The downside of the lack of sympathectomy is that it may impact the strength of analgesia and increase inter-individual variability regarding the extent of the spread of local anesthetic. Thus, the use of SAP for cardiac surgery is still in its infant stage, and more studies are needed to evaluate the effectiveness of this block, as well as to compare this block with other alternatives.

Erector spinae plane block

The erector spinae plane (ESP) block has been highlighted for its utility for thoracotomy and surgeries involving the chest and abdomen [21-23]. The benefit of the ESP block over the fascial plane blocks described above is that it has the ability to spread into ventral rami that covers T2 to T6 intercostal nerves based on cadaveric MRI assessments, which is helpful for median sternotomy [24]. This block is often referred to as “paravertebral by proxy” colloquially [25].

Krishna et al. [26] randomized 106 patients undergoing elective cardiac surgery and requiring cardiopulmonary bypass (CPB) into ESP or acetaminophen and tramadol groups. The ESP group had a significantly lower pain score (p=0.0001) and patients experienced a significantly higher duration of analgesia (p=0.0001). While there are no established guidelines, experts believe that this block should be considered superficial, and compressible in case of a hematoma [27]. Nagaraja et al. [28] randomized 50 patients undergoing cardiac surgery into bilateral continuous ESP and thoracic epidural groups. Both interventions were performed one day prior to surgery. The authors found that the duration of mechanical ventilation, incentive spirometry, and ICU stays were comparable. Even though pain scores were significantly different, mean scores for both groups were <4/10. Because the vast majority of patients undergoing cardiac surgery are anticoagulated, the thoracic epidural is a less attractive option. Results comparing ESP to thoracic epidural head-to-head show ESP to be a very attractive alternative.

Although there are theoretical risks with all blocks, such as infection, hematoma, and local anesthetic systemic toxicity (LAST), there have not been any reported complications with ESP blocks. For anticoagulated patients, the risk of neurological deficits from paraxial blocks is extremely low. The chances of epidural abscess and ipsilateral Horner syndrome are practically nil, especially compared to the neuraxial blockade. In addition, the ESP block is very easy to perform and its safety margin is very high with the use of ultrasound guidance. It is extremely rare for the needle to enter the pleural space as the goal is to have the needle contact the transverse process. On top of the safety profile, Krishna et al. [26] also found that the ESP group was extubated earlier (p=0.0001), able to tolerate a diet sooner (p=0.0001), and discharged from ICU earlier (p=0.001). Thus, the improved analgesia from ESP block not only helped with pain scores but also achieved measurable metrics that benefit the outcome of these surgeries. While the current data on ESP is very promising, this block is still very new and more studies are needed to delineate its true effectiveness in cardiac surgeries.

Paravertebral block

Paravertebral block (PVB) is a type of chest wall block that is traditionally performed via landmark-based techniques. As with most regional blocks, the ultrasound-guided approach is gaining more popularity as it becomes more widely available. The analgesic effect of this block is based on the level of the injection. However, a technique using multiple small injections where small volumes of local anesthetics are injected at several different levels is preferred over a single-shot injection [28].

Clinical data appear to be favorable towards PVB. Sun et al. [29] studied 60 patients undergoing off-pump CABG (OPCABG) comparing those with PVB combined with GA and those with GA alone. They found that pain scores, morphine-equivalent consumption, and time to extubation were lower, and ICU stay was shorter in the block + GA group. El Shora et al. [30] randomized 145 cardiac surgeries via median sternotomy into bilateral PVB + GA vs. thoracic epidural + GA. The study showed that both groups had similar pain scores, but the PVB + GA group had a shorter ICU stay (p=0.005), and lower incidence of urinary retention (p=0.04) and vomiting (p=0.018). A meta-analysis conducted by Scarfe et al. [31] reviewed 23 RCTs with 1,120 patients, comparing PVB to epidural analgesia. They also concluded that PVB was associated with a lower incidence of nausea/vomiting, urinary retention, and hypotension.

The incidence of complications after PVB is very low [28]. The benefits of PVB over thoracic epidural include more hemodynamic stability, less nausea and vomiting, and less urinary retention while maintaining adequate pain control for cardiac surgery. However, pleural puncture and pneumothorax are the two feared complications among anesthesiologists, which often dissuade us from utilizing this block.

Transversus thoracic muscle plane block

The transversus thoracic muscle plane block (TTPM) was discovered in 2015 and involves the deposition of local anesthetic in the plane between the transversus thoracic muscle and internal intercostal muscles. It anesthetizes the T2-T6 intercostal nerves [32]. In a study by Aydin et al. [33], patients undergoing median sternotomy were randomized to receive either preoperative TTPM block with 20 mL 0.75% bupivacaine or 20 mL saline bilaterally. Patients in the experimental group had reduced postoperative opioid use (p<0.001), less pain 12 hours after surgery (p<0.001), and less postoperative nausea and pruritus (p<0.001).

Zhang et al. [34] studied TTPM in 100 pediatric patients undergoing open-heart surgery, who were randomized to receive a bilateral block or no block at all. The control group was shown to have significantly higher intraoperative and postoperative fentanyl consumption. Likewise, time to extubation and lengths of ICU and hospital stay were significantly decreased in the TTPM group.

Discussion

As we move towards ERAS and stride towards opiate-free anesthesia, the world of cardiac surgery is lagging behind. The traditional heavy narcotic approach is associated with delayed extubation, urinary retention, nausea, and a variety of pulmonary issues in the perioperative period. While neuraxial techniques such as thoracic epidural, and even intentional high spinal, will provide the best analgesia for the surgery, anesthesiologists are often wary of potential adverse effects such as profound hypotension and epidural hematoma. For patients with a severe left main disease or triple vessel disease who are dependent on higher blood pressure for coronary perfusion, hypotension from neuraxial medications may cause further ischemia. In addition, these patients are often receiving heparin infusion before surgery and will be heavily heparinized intraop, and anesthesiologists may not always be willing to perform neuraxial procedures on them.

With regard to median sternotomy, PECS was better than IV analgesia in Kumar et al.'s study. However, PECS block is not known to cover the sternotomy incision dermatome since it does not cover the anterior cutaneous branches of intercostal nerves. As a result, more research is needed to delineate the mechanism of pain relief. Some case series have reported using PECS and SAP block for thoracic traumas. It does appear to be a reasonable alternative to thoracic epidurals, especially when they are contraindicated in the setting of anticoagulation. PECS and SAP can both be performed in the supine position and under general anesthesia if workflow allows, and they have a very high margin of safety as these are superficial, compressible, and far from any structure that is vulnerable to hematoma. They provide hemodynamic stability compared to neuraxial techniques such as thoracic epidurals. The major limitation of these two blocks is that they do not provide any sympathectomies, which means only somatic, not visceral analgesia. These situations can often be tricky since the patient and surgeon may be misled to believe that the block was not working.

PVB and ESP blocks do have the capability of covering the median sternotomy incision site as they are associated with the blockade of anterior branches and ventral rami of intercostal nerves. Studies have proven that PVB and ESP will have adequate coverage for median sternotomy. The American Society of Regional Anesthesia (ASRA) guidelines suggest that in the setting of anticoagulation, deep peripheral blocks are probably unsafe while superficial blocks are probably safe. Even though the ESP block has not been officially classified as deep or superficial, some experts have advocated it to be a superficial block. Currently, we are not aware of any large RCTs comparing different fascial plane blocks against each other to establish the superiority of one over the other.

## Conclusions

Regional techniques do provide significant analgesia as part of the multimodal pain management regimen. While the regional techniques mentioned above are established more outside of the cardiac realm, the available data suggest that they have tremendous potential in various types of cardiac surgeries. Since the sample sizes of the studies we reviewed are mostly very small, further studies with larger sample sizes are required to not only verify the validity of the current data but also establish the safety profiles and delineate mechanisms of the newer regional techniques.
